# Polybrominated diphenyl ether congener, BDE-47, impairs insulin sensitivity in mice with liver-specific Pten deficiency

**DOI:** 10.1186/s40608-014-0031-3

**Published:** 2015-02-07

**Authors:** Rebecca L McIntyre, Heidi L Kenerson, Savitha Subramanian, Shari A Wang, Machiko Kazami, Heather M Stapleton, Raymond S Yeung

**Affiliations:** Department of Surgery, University of Washington, 1959 NE Pacific, Seattle, WA 98195 USA; Department of Medicine, University of Washington, Seattle, WA USA; Nicholas School of Environment, Duke University, Durham, NC USA

**Keywords:** BDE-47, Insulin resistance, Pten-/- mice, Tsc1-/- mice, Metabolic disruption

## Abstract

**Background:**

The potential health effects of polybrominated diphenyl ethers (PBDEs) that are widely used as flame-retardants in consumer products have been attributed, in part, to their endocrine disrupting properties. The purpose of this study is to examine the *in vivo* effects of an early exposure to PBDEs on the development of insulin resistance in mice.

**Results:**

The metabolic consequences of BDE-47 in mice with varying insulin sensitivities secondary to liver-specific activation of Akt (*Pten*^fl/fl^;*Alb*^Cre^) and mTORC1 (*Tsc1*^fl/fl^;*Alb*^Cre^) as well as wild-type littermates, were studied. BDE-47, a dominant congener of PBDE, was given daily (1 mg/kg/day) for six weeks by oral gavage in young mice following weaning.

At the end of the exposure, there were no significant differences in total body, liver, or white adipose tissue weights between the BDE-47-treated vs. DMSO-treated mice for each respective genotype. Metabolic studies revealed significant impairment in insulin sensitivity in the BDE-47-treated *Pten*^fl/fl^;*Alb*^Cre^ mice, but not in wild-type or *Tsc1*^fl/fl^;*Alb*^Cre^ mice. This was not accompanied by significant alterations in plasma insulin levels or hepatic triglyceride accumulation in the *Pten*^fl/fl^;*Alb*^Cre^ mice. The mean plasma BDE-47 level in the wild-type mice was 11.7 ± 2.9 ng/g (wet weight).

**Conclusions:**

Our findings indicate that BDE-47 exposure during the early post-natal period induces a mild disturbance in glucose metabolism in susceptible mice with increased baseline insulin sensitivity. These results suggest an interaction between BDE-47 and genetic factors that regulate insulin signaling, which may result in long-term consequences.

## Background

Metabolic syndrome is a major health problem in developed countries and is increasing in prevalence worldwide. There are many adverse and potentially life-threatening consequences of the metabolic syndrome including atherosclerosis, diabetes, chronic liver disease, and cancer. The financial impact of these diseases is estimated to account for ~20% of all medical costs per year in the USA [1].

Genetic, behavioral and environmental factors contribute to the development of the metabolic syndrome, which is the result of the biological consequences of systemic insulin resistance. Current research suggests that perinatal and early childhood exposure to certain chemicals can disrupt endocrine response and thus, modulate the risk of obesity and metabolic syndrome in later life [2,3]. This idea is attractive, however evidence to support this hypothesis is sparse. The purpose of this study was to characterize the effects of a candidate endocrine disruptor, BDE-47, on hepatic metabolism and systemic glucose metabolism in hosts with varying susceptibilities to insulin resistance.

BDE-47 is one of the dominant congeners of polybrominated diphenyl ethers (PBDE) that are widely used as flame-retardants in consumer products [4]. These are persistent contaminants commonly found in house dust, air and soil, and their accumulation in the food chain contributes to significant levels found in humans, particularly children. Toxicologic studies have linked BDE-47 to neurotoxicity, genotoxicity, cytotoxicity, and endocrine disrupting activity. However, much less is known about its effects on glucose and lipid metabolism. Wang et al. reported that low levels BDE-47 can induce reactive oxygen species (ROS) production and lead to increased cell proliferation without evidence of DNA damage *in vitro* [5]. Here, we predict that early postnatal exposure to BDE-47 may promote insulin resistance via Akt/mTORC1 signaling.

Over-nutrition and obesity are associated with the dysregulation of the insulin signaling pathway, which leads to blunted Akt response down-stream of insulin receptor activation. Serving as a sensor and regulator of nutrient signaling, mTORC1 coordinates energy homeostasis with macro-molecular synthesis. In insulin-sensitive tissues such as the liver, mTORC1 promotes lipogenesis through the phosphorylation of lipin-1 [6,7] and dampens Akt signaling through feedback inhibition [8]. Additionally, activation of Akt in hepatocytes gives rise to steatohepatitis [9], but others and we found that hepatocytes with constitutive mTORC1 activation secondary to the loss of its negative regulator, *Tsc1*, do not have an increase in triglyceride accumulation [10,11]. Instead, we found that Akt and mTORC1 have opposing effects on hepatic lipid metabolism *in vivo*. We hypothesize that exposure at an early age to BDE-47 may alter the systemic metabolic response, which could lead to the development of metabolic syndrome. To address this experimentally, a fixed amount of BDE-47 was administered to three cohorts of young mice with varying insulin sensitivities. The results indicate that only mice showing increased insulin sensitivity (i.e., *Pten*-mutant mice) were affected by the exposure. Understanding the relationship between BDE-47 and the insulin signaling pathway is expected to have significant impact on the growing population suffering from metabolic syndrome.

## Methods

### Ethical statement

All animal experiments were performed in accordance with the Institutional Animal Care and Use Committee at the University of Washington, Seattle, under the approved protocol #3051.

### Mice

*Tsc1*^fl/fl^ mice were obtained from David Kwiatkowski at Brigham and Women’s Hostpital (Boston, MA). *Pten*^fl/fl^ (#006068) and *albumin (Alb)-Cre (#003574)* mice were purchased from Jackson Laboratories (Bar Harbor, ME). *Pten*^fl/fl^ and *Tsc1*^fl/fl^ mice have been crossed to *Alb*^Cre^ mice and subsequently intercrossed to generate *Tsc1*^+/+^;*Pten*^fl/fl^;*Alb*^Cre+^ and *Tsc1*^fl/fl^;*Pten*^+/+^;*Alb*^Cre+^ [11]. Animals with *Alb*^Cre-^ genotypes from the same litters were used as control mice. Since the transgenic mice originated from different genetic backgrounds, they have undergone multiple generations (>5) of brother-sister mating to minimize any genetic background variation. Only males were used in this study to reduce variability due to gender differences. Animals were housed 5 per cage in a modified SPF facility under 14–10 hour light/dark cycle and were fed normal chow ad libitum.

### Exposure

Certified BDE-47 was obtained form Accustandard, Inc. (New Haven, CT, No. BDE-047 N). Five mg of BDE-47 was dissolved in 250 uL of DMSO, and then further diluted into 4.75 mL of corn oil. Placebo consisted of 250 uL of DMSO diluted in 4.75 mL of corn oil. Upon weaning, 3–6 week old mice were randomly assigned to receive BDE-47 (1 mg/kg/day) or placebo for 6 weeks. For each genotype, block randomization with a block size of 4 was used to assign treatment arm. The dose (Monday through Friday) was based on daily weight measurement and the treatment was administered via oral gavage. Animals were monitored daily during treatment and were euthanized within 24 hours of the last exposure following an overnight fast.

### Glucose tolerance and insulin sensitivity testing

After four weeks of treatment, mice underwent glucose tolerance testing (GTT), followed exactly one week later by insulin sensitivity testing (IST). For GTT, mice were fasted for sixteen hours and weighed. After sixteen hours, fasting blood glucose was obtained from venous blood via tail nick and measured with the OneTouch blood glucose monitoring system and test strips from LifeScan, Inc. (Milpitas, CA). Mice received an IP injection of glucose (1 mg/g body weight). Blood glucose values were obtained at 15, 30, 60, and 120 minutes. At 30 minutes 50 μl of blood was procured via retro-orbital bleed for insulin assay. Plasma insulin was quantified using Linco Elisa Kits (Millipore, Billerica, MA).

For the IST mice were fasted for four hours and weighed. After fasting, a blood glucose level was obtained at time 0 and then 1 mU/g of insulin was given via IP injection and blood glucose values were obtained at 30, 60, 90 and 120 minutes.

### Weight measures

Following treatment, fasted mice were euthanized via anesthetized cervical dislocation. Following death, blood was drawn through cardiac puncture and tissues were harvested. Liver tissue, muscle from the quadriceps, and white adipose tissue were weighed and frozen on liquid nitrogen. Body weight, liver to body weight ratio, and white adipose tissue weight were calculated.

### Liver triglyceride analysis

Hepatic triglyceride content was determined following lipid extraction using the Folch method as previously described [11].

### BDE-47 plasma levels

Fasting plasma samples from the BDE-47-treated wild-type mice were analyzed for the concentration of the congener using established methods [12].

### Immunoblot analysis

Mouse liver tissue was homogenized in ice-cold radioummunoprecipitation (RIPA) buffer (1% Nonidet P-40, 1% sodium deoxycholate, 0.1% SDS, 0.15 M NaCl, 10 mM Tris (pH 7.2), 0.025 M β-glycophosphate (pH 7.2), 2 mM EDTA, and 50 mM sodium fluoride) with protease and kinase inhibitors (0.05 mM AEBSF, 10 μg/ml aprotinin, 10 μg/ml pepstatin, 1 mM orthovanadate, 10 μg/ml leupeptin, 1 mM microcystin LR). The protein concentration was measured using the BCA Protein Assay (Pierce, Rockford, IL). Equal amounts of protein were separated by SDS-PAGE, transferred to Immobilin-P membranes (Millipore, Bedford, MA) and blotted with antibodies from the following sources: phospho-S6 Ribosomal Protein (Ser235/236) (#2211), S6 Ribosomal Protein (#2217), phospho-Akt (Ser 473) (#4060), Akt (#9272), Tsc1 (#4906), and Pten (#9552) all from Cell Signaling (Boston, MA). Beta-Actin (#A5441) was purchased from Sigma (St. Louis, MO).

### qRT-PCR

Total RNA was extracted from liver tissue using a commercially available RNA extraction kit according to the manufacturer’s protocol (Agilent Technologies, Santa Clara, CA). After spectroscopic quantification, RNA was reverse-transcribed, and cDNA was analyzed by real-time quantitative PCR using SYBR green (600882, Agilent Technologies) master mix. Primers specific for individual genes were purchased from Invitrogen or IDT. Data were normalized to housekeeping genes Gapdh or Actb. Relative amounts of the target gene were calculated using the ΔΔCt formula.

### Statistical analyses

Based on our past studies of HFD-induced steatosis [11], a sample size of 6 mice per group has been adequate to detect a difference of 1.25 standard deviations with a confidence level of 5% and statistical power between 80% and 90%. Quantitative data were analyzed by unpaired t test. A p-value less than .05 was considered significant.

## Results

### BDE-47 did not significantly affect body weights

**Figure 1:**
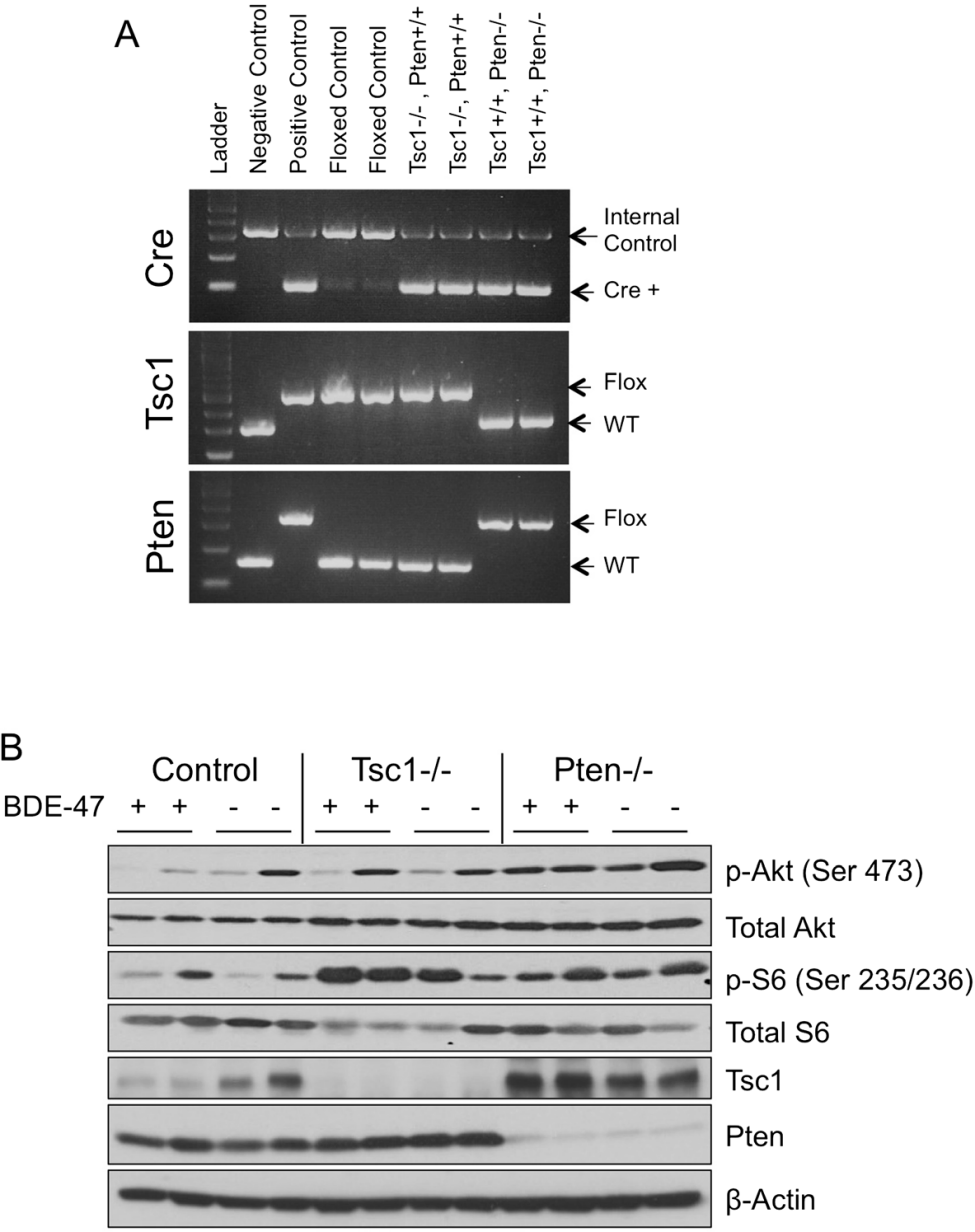
**Liver-specific ablation of** ***Pten*** **or** ***Tsc1*** **leads to increased activation of Akt or mTOR pathway. A)** PCR genotyping illustrating the detection of *Cre*, and *Tsc1* ^fl^_,_ *Pten* ^fl^ alleles in the various groups. Two of each genotype are shown. Control mice carried floxed alleles for *Tsc1* and do not possess the *Cre* allele. **B)** Western blotting of liver lysates from representative mice of each genotype using antibodies directed against the indicated proteins.

To determine the impact of BDE-47 on systemic and hepatic metabolism, mice (n = 6 per group) were randomly assigned to receive either BDE-47 (1 mg/kg/d, Monday through Friday) or vehicle (DMSO) control via oral gavage following weaning (i.e., between 3 to 6 wks old). Since the magnitude of the biologic effects of BDE-47 is not known, we chose to study mice with various sensitivities to insulin. Those with liver-specific activation of Akt (*Pten*^fl/fl^;*Alb*^Cre^, a.k.a. *Pten*−/−) are known to be hyper-sensitive to insulin, whereas those with mTORC1 activation (*Tsc1*^fl/fl^;*Alb*^Cre^, a.k.a. *Tsc1*−/−) display mild insulin resistance. Littermates harboring the floxed alleles in the absence of the *Cre* allele served as ‘wild-type’ controls. Figure 1A shows the PCR genotyping for each cohort of mice used in this study. As a result of these genetic alterations, livers from the ‘knockout’ mice showed the expected activation of Akt (illustrated by phospho-Akt(Ser473) expression) in the *Pten*−/− livers, and activation of mTORC1 (inferred by phospho-S6 (Ser235/236) expression) in the *Tsc1*−/− mice (Figure 1B).

After six weeks of treatment, the mice were fasted overnight and sacrificed via cervical dislocation under anesthesia, and tissues were harvested for analyses. Table 1 summarizes the morphometric data on liver, white adipose tissue and total body weights. No significant differences in these parameters were found between the BDE47 vs. placebo-treated groups. As previously noted, liver:body weight ratios were significantly higher among the *Pten*-mutant mice compared to the other two groups [9,11]. Plasma BDE-47 levels were determined in the wild-type mice at the end of the treatment period. Mean plasma concentration was 11.7 ± 2.9 ng/g (wet weight). Mice tolerated the treatments without any observable adverse events.

**Table 1:** **Total body, liver and white adipose tissue weights at sacrifice**

	**Control**		***Tsc1*** **−/−**		***Pten*** **−/−**	
Age	10-11 weeks		10-11 weeks		9-11 weeks	
Treatment	BDE	DMSO	BDE	DMSO	BDE	DMSO
Body weight	20.95 ± 0.22	20.01 ± 0.97	18.69 ± 0.42	19.66 ± 0.68	21.23 ± 1.51	20.92 ± 0.51
P-Value	0.6		0.4		0.9	
Liver:Body weight	3.89 ± 0.12	4.10 ± 0.26	4.27 ± 0.18	4.12 ± 0.20	4.86 ± 0.20	4.90 ± 0.13
p-value	0.5		0.6		0.86	
(BDE vs DMSO)						
p-value	0.02 (vs. Pten-/-)			0.01 (vs. Pten-/-)		
(DMSO vs. DMSO)						
White adipose tissue weight	0.243 ± 0.03	0.257 ± 0.04	0.287 ± 0.03	0.257 ± 0.03	0.238 ± 0.03	0.313 ± 0.11
p-value	0.8		0.6		0.5	
(BDE vs DMSO)						

### BDE decreases insulin sensitivity in mice with hepatocyte-specific deletion of Pten

**Figure 2:**
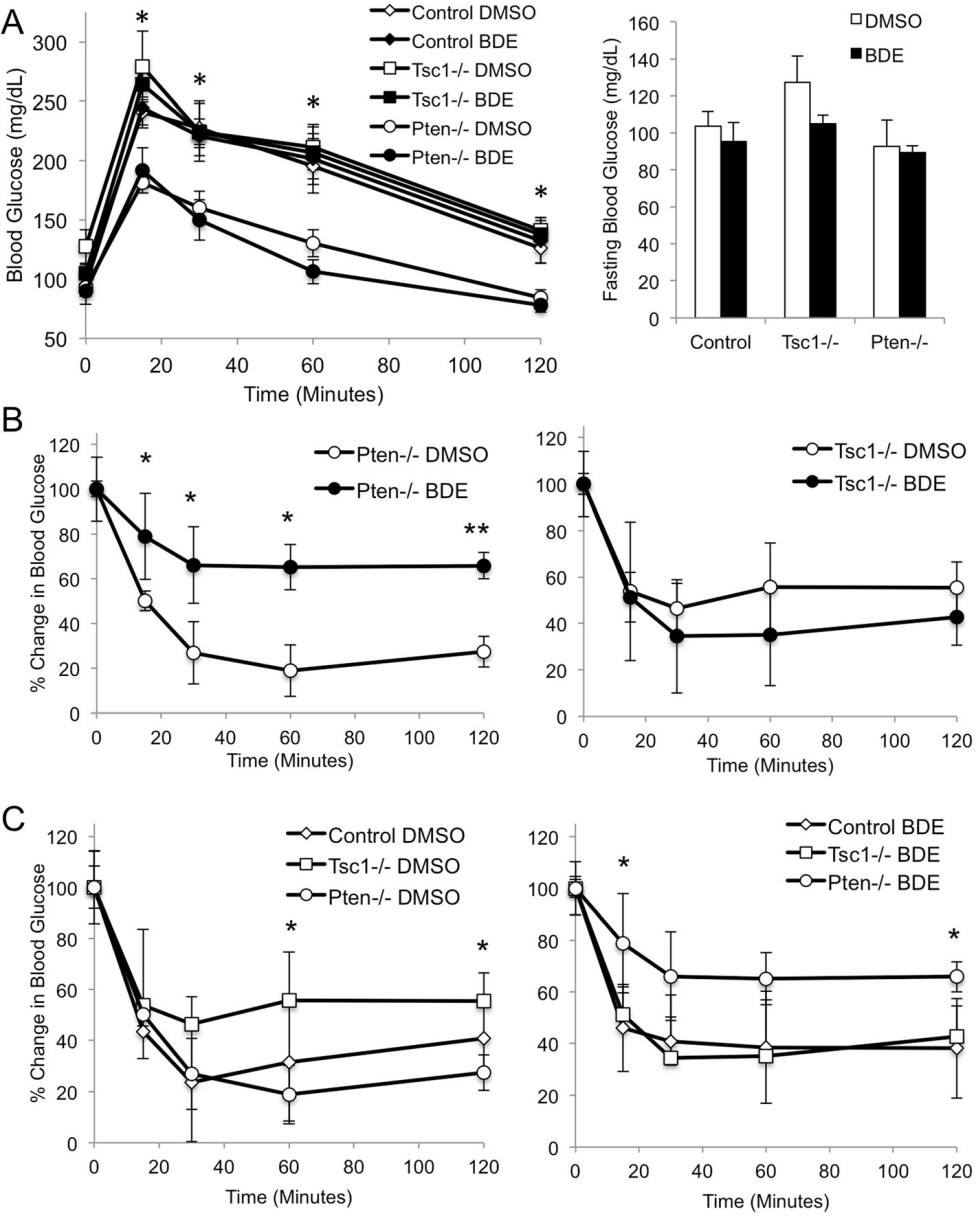
**BDE-47 reduces insulin sensitivity in mice with liver-specific** ***Pten*** **deletion. A)** Glucose tolerance of different mouse models following exposure to BDE-47 or DMSO. After an overnight fast of 16 hrs, mice were given glucose intraperitoneally (1 mg/g). Blood glucose was determined at 15, 30, 60 and 120 minutes. *, p < 0.05 *Tsc1*−/− vs. *Pten*−/− (DMSO and BDE-47 groups). **B)** Effects of BDE-47 on insulin sensitivity of *Pten*-mutant (left panel) and *Tsc1*-mutant (right panel) mice. *, p < 0.05, **, p < 0.01. **C)** Insulin sensitivity in mice of various genotypes treated with DMSO (left) and BDE-47 (right). *, p < 0.05 *Tsc1*−/− vs. *Pten*−/−.

Following 4 wks of treatment, animals underwent glucose tolerance testing followed by insulin sensitivity testing one week later. Consistent with our previous report [11], the *Pten*-mutant mice had improved glucose tolerance without a significant difference in fasting glucose concentration compared to the *Tsc1*-mutant and control mice (Figure 2A). Exposure to BDE-47 did not affect glucose tolerance but led to a significant reduction in insulin sensitivity in the *Pten*^fl/fl^;*Alb*^Cre^ mice, while the *Tsc1*^fl/fl^;*Alb*^Cre^ mice were unaffected (Figure 2B). In the DMSO-treated cohort, the *Tsc1*−/− mice were significantly less insulin sensitive than the *Pten*−/− mice as previously observed [11]. Following BDE-47 treatment, the *Pten*−/− mice became significantly more resistant to insulin compared to the *Tsc1*−/− mice (Figure 2C). Thus, glucose homeostasis was influenced by BDE-47 in a genotype-specific manner. We and others have noted that the improvement in glucose tolerance and insulin sensitivity in the *Pten*^fl/fl^;*Alb*^Cre+^ mice is a result of elevated Akt activity [11]. However, we failed to detect a consistent change in Akt(Ser473) or rpS6(Ser235/236) phosphorylation in the BDE47-treated livers to account for the observed responses to insulin in the *Pten*-mutant mice (see Figure 1B).

### Effects of BDE-47 on systemic insulin response

**Figure 3:**
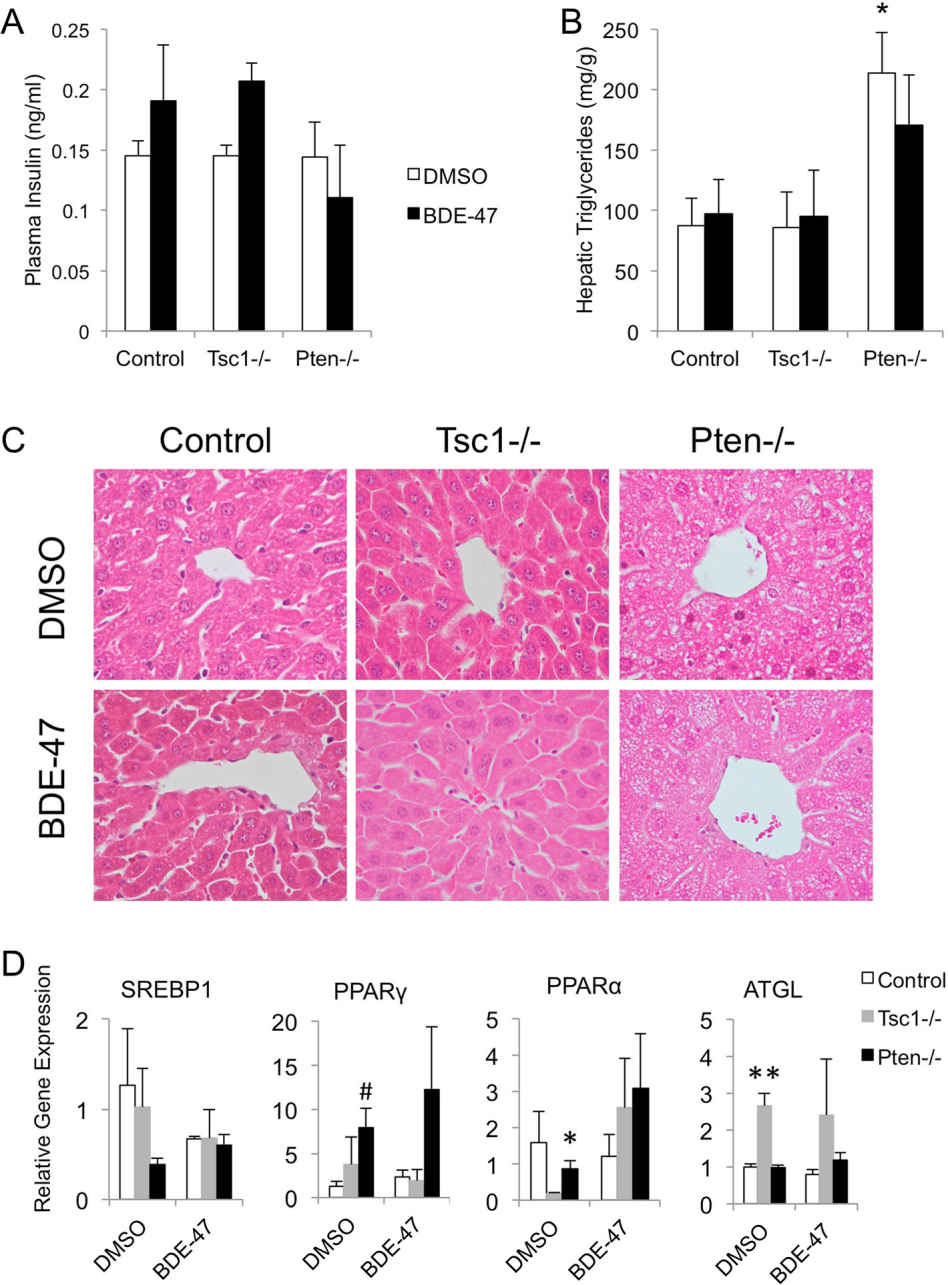
**BDE-47 does not significantly impact plasma insulin concentrations or hepatic triglyceride content. A)** Mean plasma insulin (ng/ml) determined at 30 minutes following glucose administration during GTT tests. **B)** Mean hepatic triglyceride levels (mg/g) following 6 weeks of BDE-47 or DMSO treatment. *, p < 0.05 compared to DMSO-treated control and *Tsc1*−/− mice. **C)** H&E histology of liver showing examples from each of the treated groups. Original magnification 400x. **D)** Expression of indicated genes in liver tissues based on qRT-PCR analyses. #, p < 0.05 compared to DMSO-treated control; *, p < 0.05 compared to DMSO-treated *Tsc1*−/− mice; **, p < 0.05 compared to DMSO-treated control and *Pten*−/− mice.

We surmise that the reduced insulin sensitivity along with normal glucose tolerance in the BDE-47 treated *Pten*^fl/fl^;*Alb*^Cre+^ mice could be due to an elevation in systemic insulin levels. We determined plasma insulin levels 30 minutes after glucose injection during the glucose tolerance tests. Figure 3A shows that the circulating insulin levels trended upward in the BDE-47 exposed *Tsc1*-mutant and control mice, but this did not reach statistical significance. On the other hand, no change in insulin levels was noted in the *Pten*-mutant mice following BDE-47 exposure.

### Effects of BDE-47 on hepatic triglyceride

Next, we examined the impact of BDE-47 on hepatic lipid accumulation. We hypothesized that the interaction of BDE-47 with the Akt pathway would cause increased lipogenesis within the liver. To determine this, triglyceride levels were measured using a colorimetric assay. As noted previously, *Pten*−/− mice accumulated more fat within the liver than *Tsc1*−/− or control mice [11]. However, BDE-47 treatment had no significant impact on the levels of lipid accumulation within the livers of each genotype (Figure 3B). These results were confirmed by histologic evaluation of the liver by H&E staining, which showed no excess fat accumulation in the BDE-47 treated animals compared to DMSO-treated controls (Figure 3C). Consistent with these findings, the expression of lipogenic genes, *Srebp1*, and *Pparγ*, or lipolytic gene, *Atgl*, in the liver did not change significantly with BDE-47 treatment (Figure 3C). Overall, in the *Pten*−/− mice, BDE-47 blunted the sensitivity to insulin in terms of glucose homeostasis without significant effect on lipogenesis. Additionally, there were no observed metabolic effects in the wild-type animals exposed to BDE-47. One possible explanation is that rodents have more potent peroxisomal oxidation of xenobiotic compounds in the livers compared to humans, thus diminishing the impact of the exposure. However, we found no significant difference in *Pparα* expression as a result of BDE-47 exposure (Figure 3C).

## Discussion

BDE-47 is the dominant congener of PBDEs found in human tissues, most of which originate from the widespread use of brominated flame retardants in consumer products. These compounds persist as a contaminant in house dust, air and soil, and its accumulation in the food chain combined with hand-to-mouth activity contribute to significant levels found in humans, especially children [2-4,13]. Previous studies have shown that BDE-47 can promote adipogenic differentiation through PPARγ and Akt activity *in vitro* [5,14], which support the ‘obesogen’ hypothesis. However, despite significant plasma levels of BDE-47 in our treated wild-type mice, we found no evidence of abnormal weight gain over a 6-week period as a result of BDE-47 exposure. In a model where insulin sensitivity is increased due to constitutive Akt activation in hepatocytes (i.e., *Pten*^fl/fl^;*Alb*^Cre^ mice), we documented a significant reduction in insulin sensitivity following BDE-47 exposure. This altered insulin response was not found in wild-type mice or those with intrinsic insulin resistance (i.e., *Tsc1*^fl/fl^;*Alb*^Cre^ mice); this suggests that the metabolic effects of BDE-47 are mild at a young age. The observations further highlight an *in vivo* interaction between BDE-47 and genetic factors in the development of metabolic syndrome. Following a constant exposure to BDE-47, only those with heightened insulin sensitivity were susceptible to its effects.

Given the short duration of this study, we did not observe other features of the metabolic syndrome in our BDE-47 treated cohorts such as obesity or hepatic steatosis, which may require a significantly longer period to develop while on a normal chow diet. Nonetheless, one of the key requirements in the pathogenesis of the metabolic syndrome is systemic insulin resistance, which became apparent after 5 weeks of BDE-47 exposure in the *Pten*−/− mice. Although there were no corresponding changes in glucose tolerance or circulating insulin levels, we predict that the BDE-47 treated *Pten*−/− mice would develop other signs of the metabolic syndrome later in life. Currently, we do not know if the impact of BDE-47 on insulin sensitivity persists after the exposure has ended, nor do we know the mechanism through which BDE-47 affects glucose metabolism. Our preliminary analysis of the exposed *Pten*-null livers failed to identify evidence of increased inflammation compared with untreated controls (data not shown), which has been shown to be associated with insulin resistance. The possibility of epigenetic reprogramming following early exposure to endocrine disruptors such as BDE-47 has not been explored and deserves further investigations, as do other effects of BDE-47 on adipose depots that are known to accumulate the congeners.

Studies in humans have shown consistent accumulation of PBDEs especially BDE-47 in blood and tissues, but their potential deleterious health effects remain speculative. Preclinical experiments suggest endocrine disrupting and neurodevelopmental properties that could lead to behavioral and cognitive changes in children [15]. Our findings of reduced insulin sensitivity in hosts that are hypersensitive to insulin indicate that BDE-47 can induce a measurable biologic effect in glucose metabolism. However, our study is limited by the short duration of observation and the lack of significant changes in wild-type mice. One plausible reason for the latter is due to insufficient exposure, but the plasma concentration of BDE-47 in the wild-type mice was found to be significantly higher than those reported in the average US adult population. Based on the results from the National Health and Nutrition Examination Survey (NHANES 2003–2004), US adults show a geometric mean serum concentration of 20.5 ng/g lipid for BDE-47, the most abundant of the PBDE congeners [16]. If we assume that the average lipid content of serum or plasma is 0.6%, then the median levels of BDE-47 in the US population would be ~0.12 ng/g plasma/serum; this is approximately 100x-fold less than that measured in our mice (i.e., 11.7 ng/g plasma). This suggests that changes in glucose metabolism are associated with significantly higher serum BDE-47 concentrations than those routinely found in humans. However, ~ 5% of the population has very high exposure in the range of 1,000 ng/g lipid, since BDE-47 levels follow a log-normal distribution. Thus, the BDE-47 levels in our experimental model are within clinically relevant levels, especially for those with occupational exposure to flame retardants (e.g., foam recycling, carpet padding) [17]. Another factor that can contribute to the mild effects seen in our study is the potent metabolism of xenobiotics in rodents [18], which is known to catabolize compounds such as BDE-47 with much greater capacity than humans through their peroxisomal compartment and thus reducing their metabolic impact. Consistent with this, we observed an upward, but statistically insignificant, trend in *Pparα* expression following BDE-47 treatment in the *Tsc1*−/− and *Pten*−/− livers. Our previous studies suggests that mTORC1 protects against diet-induced steatosis in part due to elevated lipolytic activity (e.g., increased *Atgl*) [11]; here, we did not challenge the BDE-47 treated *Tsc1*−/− mice with a high-fat diet to determine the extent of steatosis ‘protection’, nor did we examine the effects of BDE-47 on total body metabolism. Nonetheless, our findings indicate that early post-natal exposure to BDE-47 at relatively high concentrations induce subtle metabolic effects in young mice, which may predispose them to the consequences of metabolic syndrome in adulthood.

## Conclusions

BDE-47 treatment in early life induces insulin resistance in susceptible mice with intrinsic sensitivity to insulin. Our findings support the potential health effects of polybrominated diphenyl ether exposure in disrupting endocrine homeostasis and highlight the interaction between environmental exposure and genetic factors.
